# Incidence of Symptomatic and Asymptomatic *Leishmania donovani* Infections in High-Endemic Foci in India and Nepal: A Prospective Study

**DOI:** 10.1371/journal.pntd.0001284

**Published:** 2011-10-04

**Authors:** Bart Ostyn, Kamlesh Gidwani, Basudha Khanal, Albert Picado, François Chappuis, Shri Prakash Singh, Suman Rijal, Shyam Sundar, Marleen Boelaert

**Affiliations:** 1 Institute of Tropical Medicine, Antwerp, Belgium; 2 Banaras Hindu University, Varanasi, India; 3 B. P. Koirala Institute of Health Sciences, Dharan, Nepal; 4 London School of Hygiene and Tropical Medicine, London, United Kingdom; 5 Geneva University Hospitals and University of Geneva, Geneva, Switzerland; Institut Pasteur, France

## Abstract

Incidence of *Leishmania donovani* infection and Visceral Leishmaniasis (VL) was assessed in a prospective study in Indian and Nepalese high-endemic villages. DAT-seroconversion was used as marker of incident infection in 3 yearly surveys. The study population was followed up to month 30 to identify incident clinical cases. In a cohort of 9034 DAT-negative individuals with neither active signs nor history of VL at baseline, 42 VL cases and 375 asymptomatic seroconversions were recorded in the first year, giving an infection∶disease ratio of 8.9 to 1. In the 18 months' follow-up, 7 extra cases of VL were observed in the seroconverters group (N = 375), against 14 VL cases among the individuals who had not seroconverted in the first year (N = 8570) (RR = 11.5(4.5<RR<28.3)). Incident asymptomatic *L. donovani* infection in VL high-endemic foci in India and Nepal is nine times more frequent than incident VL disease. About 1 in 50 of these new but latent infections led to VL within the next 18 months.

## Author Summary

Visceral Leishmaniasis is well known as a public health problem in North-Indian Bihar state and adjacent districts in Nepal, with about 300.000 new cases per year. As not all infections with *L.donovani* lead to disease, the impact of control programs should not only be measured in numbers of VL cases, but also in number of new infections. So far there have been little or no data on the infection∶disease ratio for this region, and the evolution of these latent infections. Using DAT seroconversion as a marker of infection, we found incident asymptomatic infection to be nine times more frequent than incident VL disease in high-endemic villages in India and Nepal, and about 1 in 50 of these latent infections lead to VL in the next 18 months, while over 80% turn seronegative again within a year. Asymptomatic DAT-positivity detected through screening in a person with no history of VL, and especially in case of documented recent seroconversion, is a risk factor for ultimately developing VL. Further studies on transient and persistent asymptomatic *L. donovani* infection are needed to better understand their immunological patterns and serokinetics, their level of infectivity, and their potential for later progression to VL.

## Introduction

In the Indian subcontinent 200 million people are estimated to be at risk of developing Visceral Leishmaniasis (VL). VL, also known as kala-azar is a fatal parasitic disease caused by *Leishmania donovani*, an intracellular parasite transmitted in the Indian continent by *Phlebotomus argentipes*. The reported number of cases in the region was 100,000 per year in 2005, but the actual figure may be 5 to 8 times higher [1]–[3].

However, most of *L. donovani* infections remain asymptomatic [4]. Cross-sectional surveys based on serological testing by Direct Agglutination Test (DAT) [5]–[9] or ELISA [9] and/or positive delayed-type hypersensitivity (DTH) reaction to a leishmanin skin test (LST) [10]–[12] show high proportions of positive persons who never reported clinical disease. It is unclear whether these asymptomatic infected persons are infectious to the sandfly vector, whether they acquire persistent immunity or develop VL later on. The proportion of *L.donovani* infections that result in VL disease is poorly documented as this requires large prospective epidemiological studies. In Brazil the ratio of incident infection to incident disease ranged between 6.5∶1 and 18.5∶1 [13]–[15], whereas in Africa ratios ranging from 1∶2.4 to 11∶1 have been reported [16]–[19]. In the only population-based longitudinal study in South-East Asia published so far Bern *et al.* found a 4∶1 ratio of incident infection versus disease in Bangladesh [20]. Similar estimates are not yet available for India and Nepal, two other countries affected by VL in the Indian subcontinent.

The objective of this study was to examine the relationship between *L. donovani* infection and clinical disease, and to estimate the probability of progressing to clinical VL in recently infected persons in India and Nepal.

## Materials and Methods

### a. Ethical issues

A large community intervention study to measure effectiveness of long-lasting insecticide treated bednets (LN) for prevention of VL in India and Nepal (KALANET, ClinicalTrials.gov NCT00318721) provided the opportunity to document incident infection and disease in Nepal and in India. Consent for the Kalanet study was sought at three levels: community, household and individual. Communities were duly informed about the purpose of the trial and consent was sought from village leaders for inclusion of the cluster in the trial. Written informed consent was obtained from all participants or their guardians for those under 18 years old. A literate witness signed on behalf of illiterate participants who added their thumbprint to the informed consent form. Ethical clearance was obtained from the ethical committees of BHU (India), the BPKIHS (Nepal), the London School of Hygiene and Tropical Medicine (UK), and the University of Antwerp (Belgium).

### b. Study site

The study ran from November 2006 to May 2009 in 26 highly endemic villages. Selection of those villages has been described elsewhere [21], [22]. The cluster randomized controlled trial did not show any significant reduction in incidence of *L. donovani* infection or VL disease in the intervention villages compared to controls [23]. For this reason, the study subjects from both intervention and control villages were included in the current analysis.

### c. Study participants and case definitions

Cross-sectional serosurveys were conducted in November–December 2006 (M0), 2007 (M12) and 2008 (M24) in the 26 study villages. A blood sample was collected from all consenting individuals over 2 years old. Clinical follow-up was done through passive and active case detection up to May 2009, i.e. 6 months after the last serosurvey.

An incident VL case was defined as a subject with a clinical episode of VL for whom the first clinical symptoms had started after the baseline serosurvey (November 2006). From November 2006 to May 2009, all subjects with fever lasting for two weeks or more were examined by a physician and tested with a rapid diagnostic test (RDT) for VL (Kalazar Detect™ Rapid Test; InBios International, Seattle, WA). Most VL cases were managed free of charge at the reference treatment centers i.e. Kala-azar Medical Research Center, Muzaffarpur, India (KAMRC) and B.P. Koirala Institute of Health Sciences, Dharan, Nepal (BPKIHS) where diagnosis was further confirmed by direct microscopic examination and/or culture of bone marrow or spleen tissue aspirate. For those who were diagnosed and treated outside the two reference centers details on diagnosis and treatment were collected from the subject or his family, and double-checked with clinical records.

Direct agglutination test (DAT) was used as a marker of *L.donovani* infection. We used a cut-off titre of 1∶1600 to define seropositivity. This cut-off is lower than the one used for VL diagnosis in clinical suspects (1∶3200) because we wanted to increase the sensitivity to detect *L. donovani* infection [24], [25]. Seroconversion was defined as a titer increase of 2 titres or more above his/her baseline value, since a difference of one titer is considered a very common (and accepted) inter-observer discrepancy in routine DAT serology reading [26].

### d. Laboratory procedures

In each serosurvey, capillary blood samples were collected from all participants on Whatman 3 filter paper. DAT was performed in the laboratories of Banaras Hindu University (BHU -Varanasi) for India, and in BPKIHS for Nepal, using a freeze-dried version of DAT antigen composed of fixed, trypsin-treated and stained promastigotes of *L.donovani* prepared in ITM-A [27]. Ten per cent of the samples were repeated in the partner laboratory for quality control. DAT was conducted as described by Harith *et al.* [28]. Briefly, disks of 5 mm diameter containing 50 µl of blood were punched out of the filter paper and eluted overnight in a 1000 µl tube at 4°C in Phosphate Buffered Saline (PBS – 7.2) supplemented with protein, to obtain a starting dilution of 1∶400. Serial dilution of the eluate in 0.9% saline, 1% foetal bovine serum and 0.24 ml 2-mercaptoethanol were made in V-shaped well microtiter plates, giving a range from 1∶400 up to 1∶25600. Results were read visually against a white background after 18 h.

### e. Data analysis

In order to allow comparison of our results with publications from other VL endemic areas we calculated the relation between infection and disease in two ways: 1) as a ratio of number of incident infections to incident VL cases in a given time period, and 2) as a rate ratio.

The ratio of infection to clinical disease was calculated comparing the number of asymptomatic seroconversions with the number of incident VL cases in a given period.

The ratio of the infection rates was calculated using the incidence rates of infection (seroconversion) and clinical VL over the available time period of observation (2 years for incident infection, two and a half years for incident VL).

Risk of progressing to clinical VL in recently infected asymptomatic persons was calculated by comparing the cumulative incidence of VL in the one and a half year of follow-up in the cohort of seroconverters of year 1, with the cumulative incidence of VL in those who had not seroconverted in the same year 1.

We also calculated two other incidence rates of interest: i) the incidence rate of VL over the 2.5 years in the full population (instead of the cohort with full serological data only), and ii) the incidence rate of VL in year 1 in those who were DAT-positive at baseline.

Confidence intervals were calculated using 95% CI formula for rates, and Pearson's χ^2^ for CI on rate ratios.

The Kalanet study was funded by the European Commission (contract No INCO-CT 2005-01537, Kalanet project). The funders had no role in study design, data collection and analysis, decision to publish, or preparation of the manuscript.

## Results

**Figure 1:**
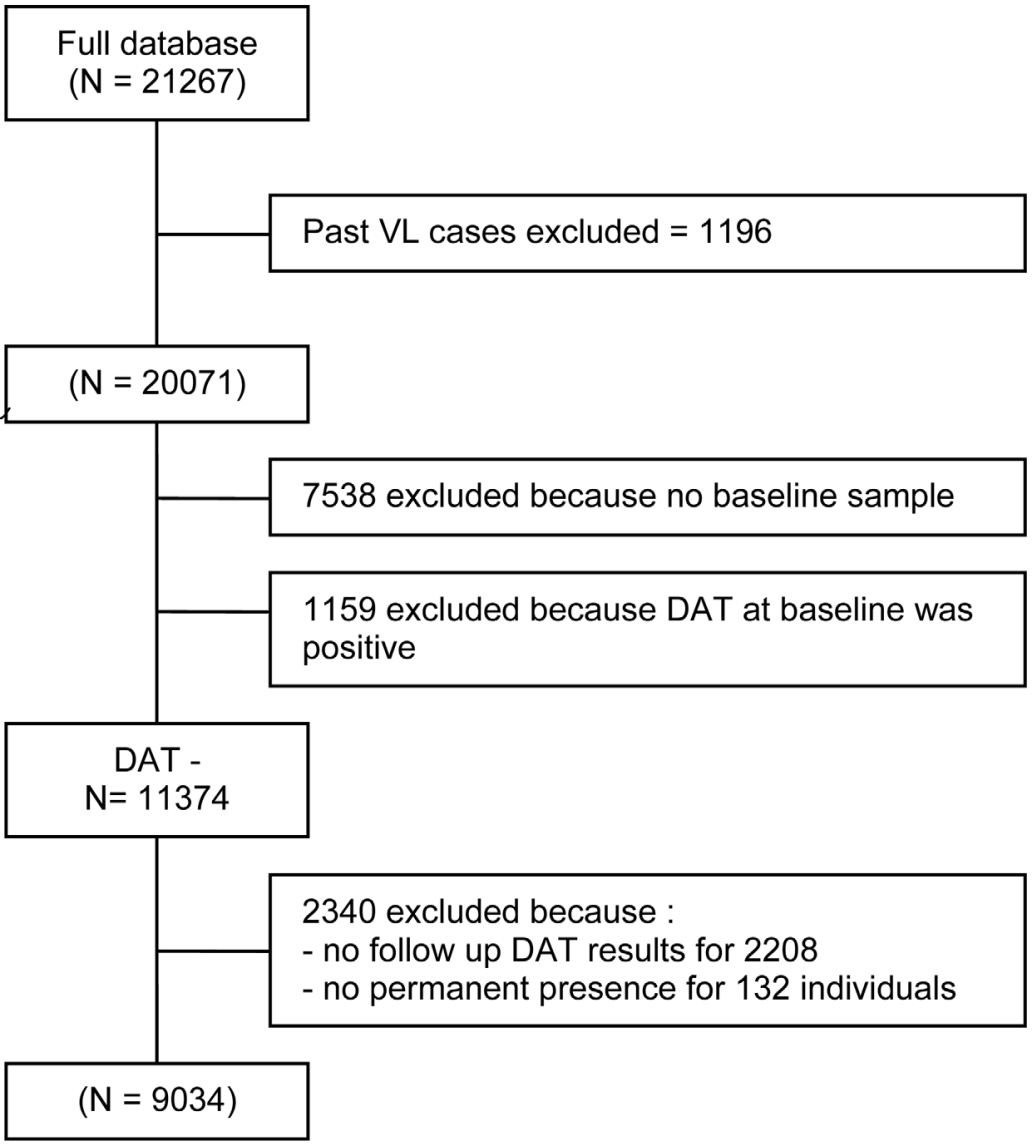
Flowchart of selection process for study population. Population that was followed up during the Kalanet study covered 21,267 individuals in 26 highly endemic villages in India and Nepal. For the current analysis, only individuals who 1) had no history of Visceral Leishmaniasis 2) were seronegative at baseline (DAT-) and 3) had complete clinical and serological data from baseline to end of the study period = 30 months. DAT = Direct Agglutination Test, cut-off used for positivity was titer ≥1∶1600.

The total study population was 21,267 of which 49.1% were women; 17.0% were under five years of age, 42.6% under fifteen. Details on the study populations in India and Nepal are provided elsewhere [21]–[23]. In brief, individual (i.e. age and gender distribution) and socio-economic characteristics were similar in both countries. The main difference was that the prevalence of DAT-positive results in those with no VL history at baseline was almost twice as high in India as it was in Nepal (18 vs 9%). In the study population with no VL-history (N = 20071) and regardless of serostatus, 120 new VL cases were diagnosed during the study (VL relapses excluded), or an overall incidence rate of VL of 2.4/1000 PY (95% CI 1.9–2.9). 1,196 had had VL in the past and 90.6% of them were DAT positive. From those without prior VL and who provided a sample at baseline, 1,159 (9.25%) were DAT-positive and 11,374 (90.75%) were DAT-negative. A cohort of 9,034 baseline DAT-negative individuals with full clinical and serological dataset was retained for the infection∶disease ratio analysis, and for the calculation of the incidence rate of seroconversion. Participants' flow (exclusion/inclusion) is summarized in Figure 1.

**Figure 2:**
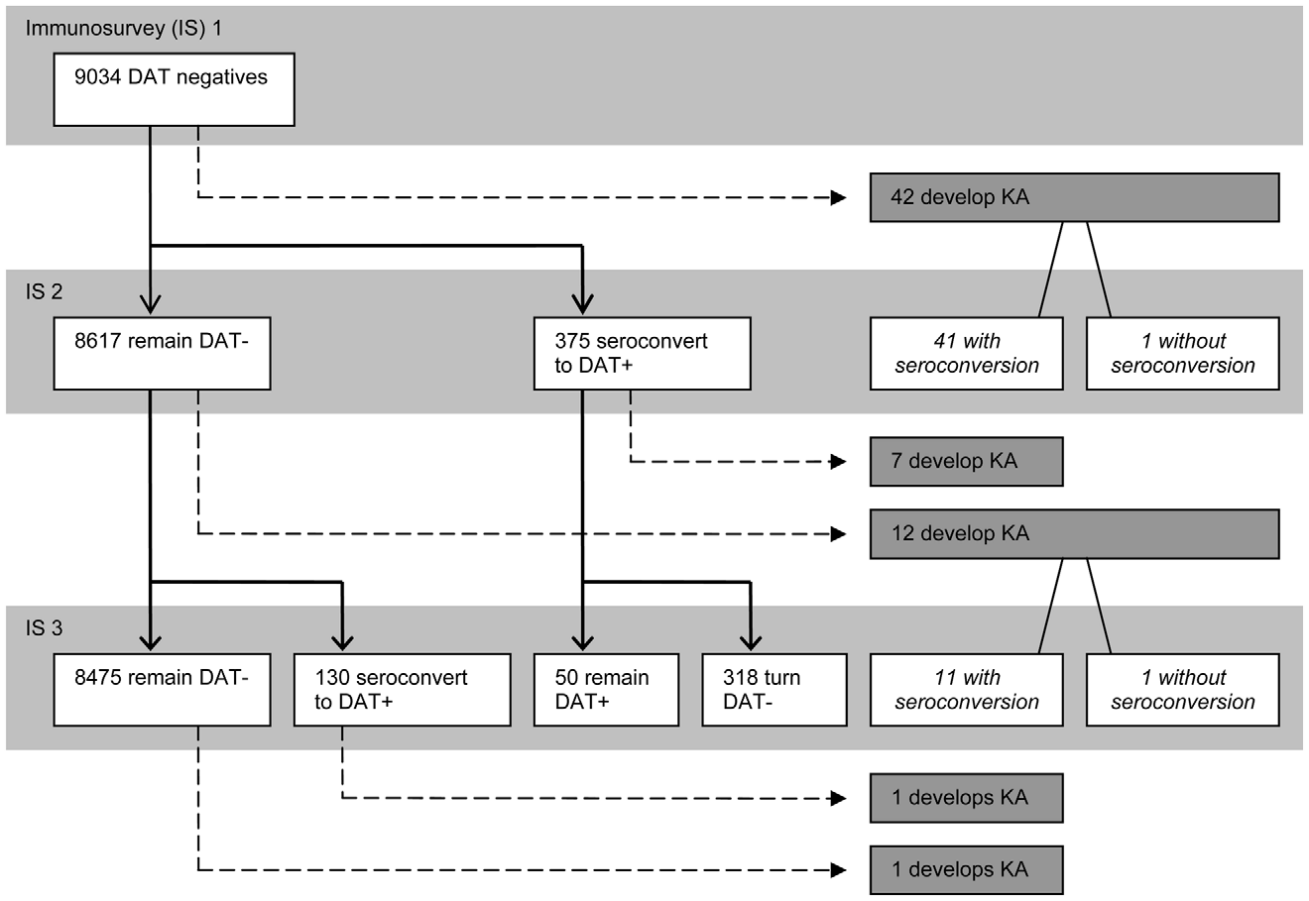
Flowchart of clinical and serological events in study population. Bars in light grey: Results of the 3 serosurveys IS1 (Immunosurvey 1), IS2 and IS3 that took place in November–December of 2006, 2007 and 2008 respectively. In between the bars: clinical events occurring in between the serosurveys, or in the 6 months following the last serosurvey.

Clinical and serological outcomes of the 9034 DAT-negative persons at baseline are detailed in Figure 2. Sixty three persons developed VL in the course of the study, 42 in the first year (Nov 2006 to Oct 2007), 19 in the second (Nov 2007 to Oct 2008) and 2 in the six months of follow up after the last serosurvey (Nov 2008 to May 2009) giving an average incidence rate of 2.8/1000 person years (PY) (95% CI: 2.1–3.5). Of the 61 VL cases occurring before the final serosurvey (Nov 2008), 59 had a documented seroconversion while 2 remained DAT-negative. These 2 cases were young children (5 and 6 years old) suffering from malnutrition. Both cases had a RDT positive test at the time of diagnosis but DAT-negative results at the serosurveys that preceded and followed their VL episode.

All calculations of incidence rates, per year, for disease and infection are shown in Table 1. Results per country are provided in Table S1.

**Table 1:** Rate and ratio calculations per year.

		Cases	Denom.	Rate/1000PY (95% CI))
Year 1	VL incidence year 1	42	9034	4.65 (3.24–6.06)
	Seroconversion incidence during year 1	416		46.05 (41.62–50.47)
	Asymptomatic seroconversion incidence	375		41.51 (37.31–45.71)
Year 2	VL incidence in 18 months of follow up	21	8986	1.56 (0.89–2.23)
	- in seroconvertors of year 1	7	375	12.52 (3.25–21.80)
	- in DAT-negatives	14	8570	1.08 (0.52–1.65)
	VL incidence during year 2 (12months)	19	8992	2.11 (1.16–3.06)
	Seroconversion incidence during year 2	141	8617	16.36 (13.66–19.06)
TOTAL	VL incidence in 30 months of follow-up	63	22512,5	2.80 (2.11–3.49)
	Seroconversion incidence	557	17651	31.56 (28.94–34.18)
	Asymptomatic seroconversion incidence	505	17651	28.61 (26.11–31.11)
Ratio infection∶ disease:				
	- year 1		8.9 ∶ 1	
	- year 2		10.8 ∶ 1	
	- full study period		8.0 ∶ 1	
Rate ratio infection ∶ disease:				
	- year 1		9.9 ∶ 1	
	- year 2		11.7 ∶ 1	
	- full study period		11.3 ∶ 1	

*Definitions: Asymptomatic seroconversion: Seroconversion in DAT titer compared to previous year's DAT result, without clinical signs of VL at the time of the DAT-positive blood sampling.*

In the first year, 42 individuals in the cohort developed VL, 375 showed an asymptomatic seroconversion and 8617 kept their seronegative status. The infection∶disease ratio was thus 8.9 to 1 (Table 1).

In the follow-up period (18 months), 7 of the 375 latently infected individuals developed VL, giving an incidence rate of 12.44/1000 PY (95% CI: 3.25–21.80) in this group. In the 8617 with no seroconversion or VL recorded in the first year, 14 new VL cases occurred in the follow-up period, giving an incidence rate of 1.1/1000 person years (95% CI: 0.23–1.94). By the time of the third serosurvey (November–December 2008), 85% (318) of the asymptomatic seroconverters had turned seronegative again.

In year 2 (12 months' period) the infection∶disease ratio was 130 asymptomatic seroconverters against 12 new VL cases, or 10.8∶1. The subsequent follow-up period (6 months) was too short to calculate VL incidences in this group of seroconverters.

Incidence rate of DAT seroconversion was 46.0 per 1000 person-years (PY) in the first year (95% CI: 41.6–50.5) and 16.4 per 1000 PY in the second (95% CI 13.7–19.1). Over the two years, the average infection incidence rate was 31.6/1000 PY (95% CI 28.9–34.2).

Incidence rate of VL was 4.6/1000 PY in the first year (95% CI 3.2–6.1) and 2.1/1000 PY in the second (95% CI 1.2–3.1). The average incidence rate of VL over 30 months in this cohort of 9034 was 2.8/1000 PY (95% CI, 2.1–3.5). The ratio between the incidence rates of infection and disease in the cohort was thus 11.3 to 1 (31.6 divided by 2.8).

Recent seroconversion was a strong risk factor for the development of VL in these high endemic villages. Incidence of VL in year 2 in persons with recent seroconversion was 11.5 times higher than in the rest of the village population (12.4 vs 1.1/1000 PY = 4.66<RR<28.30).

Incidence of VL in year 1 in those who were already DAT positive at baseline (data not shown here) was 15.9/1000 PY (95% CI 8.5–23.2), more than 3 times the 4.6/1000 PY incidence rate found in year 1 in individuals with negative DAT at baseline (Relative risk 3.42 (1.97<RR<5.92; p<0.0001)).

## Discussion

Prospective epidemiological population-based studies on incidence of *L. donovani* infection and VL disease are scarce. This is the first study done in India and Nepal providing data on infection∶disease ratio and on the risk for development of VL in recently infected individuals.

In our study, the ratio of numbers of infection to disease was about 9 (8.9 for the first year, 8.0 over the whole study period) and the rate ratio about 10 (9.9 for year 1 and 11.3 for the full length of the study). Because of the relatively low number of VL cases, calculations per country per year give a wider variation. Nevertheless, incidence rates of infection and disease in India are generally twice as high as in Nepal, and in both countries rates decreased in the second year (Table 1). The ratio infection∶disease was 7.6 for India and 9.6 for Nepal, and rate ratios were 10.8 and 13.2 respectively.

The ratio of infection to disease may differ from one village to another and also fluctuates over time within the same population, as reported earlier by Khalil *et al.* [29]. The determinants of this ratio include age, genetics, nutritional status and concomitant disease, all features that can be clustered in villages or parts of villages. The most important determinant appears to be living in proximity to a previous VL patient, as shown in a recent review by Bern *et al.* [30]. The closer to a VL case, the higher is the probability of infection becoming symptomatic.

Similar studies have been conducted in VL-endemic areas in Brazil, Ethiopia, Kenya, Sudan and Bangladesh, but methods differ between studies (i.e. in terms of serological tests and case definitions), and are sometimes not explicitly stated. Most studies report the ratio of asymptomatic infection to disease. These ratios range from 18.5∶1 to 6.5 in Brazil (American VL occurring in children below 15 and caused by *L. infantum/chagasi*) [13], [15], and from 11∶1 to 1∶2.4 in Africa [18], [29]. In other prospective studies, incidence rates of infection (seroconversion) and VL disease are reported, allowing to calculate the Rate Ratio (RR): these rates were respectively 46/1000 and 5.1/1000 PY (RR = 9.0) in Brazil [13], 9/1000 and 2.2/1000 PY (RR = 4.1) in Baringo district, Kenya [17] 106/1000 and 19.4/1000 PY (RR 5.5∶1) in Ethiopia [16] and 63.1/1000 and 15.6/1000 PY (RR = 4∶1) in Bangladesh [20].

Our analyses were limited in different ways. In contrast with the aforementioned studies, we only used one serological test (DAT) and did not test for cellular immunity (LST). Studies on asymptomatic *L.donovani* infection are hampered by the absence of validated definitions and appropriate biological markers. The pathway, starting from the initial infective sandfly bite up to symptomatic visceralisation or asymptomatic infection is extremely complex, whereby different types of immunity (innate, humoral, cellular) play their role in the process of clearing or containing the progressive infection in skin, lymph node and spleen [31] : It can be assumed that in a number of individuals infection will be cleared rapidly by innate immunity, not resulting in an over time measurable immune response, while in the other extreme the infection may only be contained at the latest stage, by (long-lasting) cell-mediated immunity after visceralisation. In comparative studies of serologic tests for asymptomatic VL, the different tests validated for confirmation of symptomatic VL (DAT, immunofluorescence (IFAT), ELISA using promastigotes as well as recombinant antigen, and LST) and comparison with PCR methods show low agreement [32]–[34] suggesting that different tests may indicate different time points and/or levels of infection/immune responses), and that only a combination of different tests can pick up all new infections. We chose DAT because it is relatively cheap and easy to perform [28], and because in comparison to LST, it requires only one visit and seroconversion occurs early after infection. A positive LST result is thought to indicate durable cell-mediated immunity after asymptomatic infection or clinical cure of VL, but LST positivity may only appear months to years after infection. A problem is that in LST positives, transient humoral immune responses can still be measured [35], be it as a sign of re-exposure, or a change in the capacity of the host to control the latent infection. A number of DAT seroconversions observed in our study may thus be reactivations in individuals with already acquired cellular immunity, and may not represent a risk to develop disease. In the Bangladesh study, the LST prevalence was 33% [20]. Earlier studies in high-endemic foci in India and Nepal give prevalence figures of 19% and 13.2% [5], [36] respectively. In the preparation phase of the KALANET study, LST performance was evaluated (*L. major* antigen, Pasteur Institute, Iran) but gave unreliable results [37] and was therefore abandoned.

The incidence of infection was measured by yearly serological testing. In the observed seroconverters who remained asymptomatic, we found that seropositivity did not last into the second year in 86.7%. This is in line with what is reported elsewhere [7], [13], [17]. Six-monthly serological testing might therefore have detected more seroconversions, which would have led to a higher ratio.

In the 375 DAT-seroconverters that were asymptomatic at the time of serodiagnosis, 7 developed VL (almost 1 in 50), showing an increased risk compared to non-seroconverters. Recent seroconversion in DAT must therefore be considered as a risk factor for VL. In a prospective study done in 2005–07 in a single community of Muzaffarpur district, Bihar State, India [9], no statistical difference in VL incidence had been observed between those DAT-positive and -negative at baseline in the 2 years following the survey, but serostatus was only tested once (at baseline) giving no data on recent seroconversion. In other, smaller studies, DAT positivity was a strong predictor of developing VL but these studies were done in asymptomatic household contacts of active VL and PKDL patients [38], [39] where VL incidence is known to be high [40]. In a recent study in Bihar by Topno *et al.* [41] 18.42% of the 38 healthy individuals who tested positive to either DAT, rK39 strip test or PCR developed clinical VL in less than 6 months. The rate of progression to disease reported was 17.85/1000 PY which is similar to the one we obtained in DAT positive individuals at baseline (15.9/1000 PY). In contrast to our study, the Topno *et al.* study was based on a small cohort (n = 335) from a single village and asymptomatic cases, identified in a single cross-sectional survey, were followed for a maximum of 12 months.

For those ultimately evolving to clinical VL, DAT may become positive some months before clinical symptoms appear [42]. By timing our yearly serosurveys in November and December, which is shortly after the pre-winter peak of sandfly density [43], we may have picked up more pre-clinical VL cases than other studies. Incubation period for full-blown visceral leishmaniasis is typically 3–8 months [19]. Prospective studies, including ours, show disease conversion in DAT-positive individuals taking place at any time, mostly within 6 months [7] but also up to two years after the baseline survey [9]. Analysis of all incident VL cases in our study has shown that 35% of them were already DAT-positive in the serosurvey that pre-ceded the year of developing VL (data not reported here). From those who developed VL in the 2^nd^ and 3^rd^ year of the study and had baseline DAT results (resp. 27 and 5), 20% were already seropositive at baseline.

PCR-detection of the parasites could have been an alternative method to estimate the infection rates. In a recent study done on a subgroup of the study population [44], we found similar positivity rates between PCR and DAT (respectively 18 and 16.1%), but a poor agreement between the two methods. This highlighted the specific character of both methods to explore asymptomatic infections, (i) PCR being probably more informative to detect very recent infections, before any immune response has controlled the infection, (ii) while DAT provides a later picture of the infections. Follow-up studies are needed to further precise the dynamics of the infection markers revealed by both methods (including the return to negative status over time) but in the meantime, DAT appears more adequate, especially for high-throughput applications.

There was a sharp decline in the incidence of VL in year 2 compared to year 1. Several explanations can be put forward: VL occurs in localized epidemics so when villages were selected on the basis of VL incidence, they were likely to be at their peak incidence after which incidences were likely to decrease. One may also hypothesize that the active and systematic screening for suspected cases and subsequent treatment may have had an impact on transmission. On the other hand, a decline in VL incidence has been observed in the national records of Nepal and India over recent years, expressing possibly the result of increased control efforts, alongside other factors such as climate changes influencing sandfly density.

### Conclusion

Using DAT seroconversion as a marker of infection, we found incident asymptomatic infection to be eight times more frequent than incident VL disease in India and Nepal, and about 1 in 50 of these latent infections lead to VL in the next 18 months.

Asymptomatic DAT-positivity detected through screening in a person with no history of VL, and especially in case of documented recent seroconversion, is a risk factor for ultimately developing VL. Further studies on latent infection are needed to better understand the serokinetics, and possibly identify markers for progression to VL. Such studies should ideally combine DTH tests, PCR and different serological tests to measure levels and timing of humoral and cellular response after exposure.

## Supporting Information

Table S1 **Additional information to the results presented in** **Table 1** **.** Rate and ratio calculations per year and by country. *Definitions: Asymptomatic seroconversion: Seroconversion in DAT titer compared to previous year's DAT result, without clinical signs of VL at the time of the DAT-positive blood sampling.*
(DOC)

Checklist S1 STROBE Checklist.
(DOC)
