# Supplementary material for: Incidence of Symptomatic and Asymptomatic Leishmania donovani Infections in High-Endemic Foci in India and Nepal: A Prospective Study
Source: PLoS Negl Trop Dis. 2011 Oct 4;5(10):e1284. doi: 10.1371/journal.pntd.0001284 (PMC3186756; doi:10.1371/journal.pntd.0001284)
Supplement: Table S1 — Additional information to the results presented in Table 1 . Rate and ratio calculations per year and by country. Definitions: Asymptomatic seroconversion: Seroconversion in DAT titer compared to previous year's DAT result, without clinical signs of VL at the time of the DAT-positive blood sampling. (DOC) [file pntd.0001284.s001.doc]

Table 1: Rate and ratio calculations per country and per year

|  |  |  | All | | |  | India | | |  | Nepal | | |
| --- | --- | --- | --- | --- | --- | --- | --- | --- | --- | --- | --- | --- | --- |
|  |  |  | Cases | Denom. | Rate /1000PY (95% CI)) |  | Cases | Denom. | Rate /1000PY (95% CI) |  | Cases | Denom. | rate /1000PY (95% CI) |
| Year 1 | VL incidence year 1 |  | 42 | 9034 | 4.65 (3.24 – 6.06) |  | 35 | 5146 | 6.80 (4.55 – 9.05) |  | 7 | 3888 | 1.80 (0.47 – 3.13) |
|  | Seroconversion incidence during yr 1 |  | 416 |  | 46.05 (41.62 – 50.47) |  | 308 |  | 59.85 (53.17 – 66.54) |  | 108 |  | 27.78 (22.54 – 33.02) |
|  | Asymptomatic seroconversion incidence |  | 375 |  | 41.51 (37.31 – 45.71) |  | 274 |  | 53.25 (46.94 – 59.55) |  | 101 |  | 25.98 (20.91 – 31.04) |
| Year 2 | VL incidence in 18 months of follow up |  | 21 | 8986 | 1.56 (0.89 – 2.23) |  | 15 | 5107 | 1.96 (0.97 – 2.95) |  | 6 | 3879 | 1.03 (0.21 – 1.86) |
|  | - in seroconvertors of year 1 |  | 7 | 375 | 12.52 (3.25 – 21.80) |  | 5 | 274 | 12.24 (1.51 – 22.97) |  | 2 | 101 | 13.29 (-5.13 – 31.71) |
|  | - in DAT-negatives |  | 14 | 8570 | 1.08 (0.52 – 1.65) |  | 10 | 4799 | 1.38 (0.52 – 2.23) |  | 4 | 3771 | 0.71 (0.01 – 1.40) |
|  | VL incidence during year 2 (12months) |  | 19 | 8992 | 2.11 (1.16 – 3.06) |  | 13 | 5111 | 2.54 (1.16 – 3.93) |  | 6 | 3881 | 1.55 (0.31 – 2.78) |
|  | Seroconversion incidence during yr 2 |  | 141 | 8617 | 16.36 (13.66 – 19.06) |  | 113 | 4837 | 23.36 (19.05 – 27.67) |  | 28 | 3780 | 7.41 (4.66 – 10.15) |
| TOTAL | VL incidence in 30 months of follow-up |  | 63 | 22512,5 | 2.80 (2.11 – 3.49) |  | 50 | 12806 | 3.90 (2.82 – 4.99) |  | 13 | 9706,5 | 1.34 (0.61 – 2.07) |
|  | Seroconversion incidence |  | 557 | 17651 | 31.56 (28.94 – 34.18) |  | 421 | 9983 | 42.17 (38.14 – 46.20) |  | 136 | 7668 | 17.74 (14.76 – 20.72) |
|  | Asymptomatic seroconversion incidence |  | 505 | 17651 | 28.61 (26.11 – 31.11) |  | 380 | 9983 | 38.06 (34.24 – 41.89) |  | 125 | 7668 | 16.30 (13.44 – 19.16) |
|  |  |  |  |  |  |  |  |  |  |  |  |  |  |
| Ratio infection : disease | |  |  | Ratio |  |  |  | Ratio |  |  |  | Ratio |  |
|  | - year 1 |  |  | 8.9 : 1 |  |  |  | 7.8 : 1 |  |  |  | 14.4 : 1 |  |
|  | - year 2 |  |  | 10.8 : 1 |  |  |  | 13.2 : 1 |  |  |  | 6.0 : 1 |  |
|  | - full study period |  |  | 8.0 : 1 |  |  |  | 7.6 : 1 |  |  |  | 9.6 : 1 |  |
| Rate ratio infection : disease | |  |  |  |  |  |  |  |  |  |  |  |  |
|  | - year 1 |  |  | 9.9 : 1 |  |  |  | 8.8 : 1 |  |  |  | 15.4 : 1 |  |
|  | - year 2 |  |  | 11.7 : 1 |  |  |  | 14.1 : 1 |  |  |  | 7.0 : 1 |  |
|  | - full study period |  |  | 11.3 : 1 |  |  |  | 10.8 : 1 |  |  |  | 13.2 : 1 |  |

*Definitions: Asymptomatic seroconversion: Seroconversion in DAT titer compared to previous year’s DAT result, without clinical signs of VL at the time of the DAT-positive blood sampling*
